# Five new *Platocoelotes* species (Araneae, Agelenidae) from caves in southern China

**DOI:** 10.3897/zookeys.512.9989

**Published:** 2015-07-06

**Authors:** Lu Chen, Shuqiang Li, Zhe Zhao

**Affiliations:** 1 College of Life Sciences, Hebei University, Baoding, Hebei 071002, China; 2 Institute of Zoology, Chinese Academy of Sciences, Beijing 100101, China

**Keywords:** Taxonomy, Coelotinae, description, diagnosis, etymology

## Abstract

Five new *Platocoelotes* species are described based on both sexes collected from caves in southern China. They are: *Platocoelotes luoi* **sp. n.** from Jiangxi, *Platocoelotes qinglinensis* **sp. n.** from Yunnan, *Platocoelotes shuiensis* **sp. n.** from Guizhou, *Platocoelotes tianyangensis* **sp. n.** from Sichuan and *Platocoelotes xianwuensis* **sp. n.** from Hubei.

## Introduction

The spider genus *Platocoelotes* was established by Wang (2002) for one coelotine from Hunan, China: *Coelotes impletus* Peng & Wang, 1997. Additionally, Wang (2003) described one new species: *Platocoelotes kailiensis* Wang, 2003 and revised three species: *Platocoelotes impletus* (Peng & Wang, 1997), *Platocoelotes icohamatoides* (Peng & Wang, 1997) and *Platocoelotes lichuanensis* (Chen & Zhao, 1998) that were transferred from the genus *Coelotes*, and revised three species in detail: the species diagnosis and the descriptions of epigynes and male palps. Currently, there are seventeen valid *Platocoelotes* species, sixteen of which are known from southern China and one, *Platocoelotes uenoi* (Yamaguchi & Yaginuma, 1971), is from Japan (World Spider Catalog 2015).

This paper provides descriptions of five new *Platocoelotes* species collected from caves in southern China. Three of them, *Platocoelotes qinglinensis* sp. n., *Platocoelotes shuiensis* sp. n. and *Platocoelotes tianyangensis* sp. n., have simple, looped spermathecae, indistinct copulatory ducts, distinct epigynal hoods, and a slender anterior apophysis, they are congeneric with *Platocoelotes ampulliformis* Liu & Li, 2008, *Platocoelotes brevis* Liu & Li, 2008, *Platocoelotes latus* Xu & Li, 2008, *Platocoelotes paralatus* Xu & Li, 2008 and others, so these three new species are easily classified as *Platocoelotes*. The other two new species have the main characters: a posterior conductor apophysis on the male palp, the presence of a large atrium, and the absence of epigynal teeth on the female epigyne, which indicate that they are congeneric with the type species of *Platocoelotes*.

## Material and methods

Specimens were examined with a LEICA M205C stereomicroscope. Images were captured with an Olympus C7070 wide zoom digital camera (7.1 megapixels) mounted on an Olympus SZX12 dissecting microscope. Epigynes and male palps were examined after dissection from the spiders’ bodies.

All measurements were obtained using a LEICA M205C stereomicroscope and are given in millimeters. Leg measurements are shown as: Total length (femur, patella + tibia, metatarsus, tarsus). Only structures (palp and legs) of the left body side were described and measured. The terminology used in the text and the figure legends follows Wang (2002). Abbreviations used in this paper and in the figure legends: A = epigynal atrium; ACA = anterior conductor apophysis; ALE = anterior lateral eye; AME = anterior median eye; AME-ALE = distance between AME and ALE; AME-AME = distance between AME and AME; ALE-PLE = distance between ALE and PLE; CD = copulatory duct; CDA = dorsal conductor apophysis; CF = cymbial furrow; E = embolus; EB = embolic base; FD = fertilization duct; H = epigynal hood; LTA = dorso-retrolateral tibial apophysis; OC = outgrowth of anterior conductor apophysis; PA = patellar apophysis; PCA = posterior conductor apophysis; PLE = posterior lateral eye; PME = posterior median eye; PME-PLE = distance between PME and PLE; PME-PME = distance between PME and PME; RTA = retrolateral tibial apophysis; S = spermatheca; SH = spermathecal head; SL = spermathecal lobe; SST = spermathecal stalk; ST = subtegulum; T = tegulum; VPA = ventral patellar apophysis.

A partial fragment of the mitochondrial gene cytochrome oxidase subunit I (COI) was amplified and sequenced for *Platocoelotes luoi* sp. n., *Platocoelotes qinglinensis* sp. n., *Platocoelotes shuiensis* sp. n., *Platocoelotes tianyangensis* sp. n. and *Platocoelotes xianwuensis* sp. n. following the protocol in Miller et al. (2009). Primers used in this study are: LCO1490 (5’-CWACAAAYCATARRGATATTGG-3’) (Folmer et al. 1994) and HCO2198zz (5’-TAAACTTCCAGGTGACCAAAAAATCA-3’) (this study). All sequences were blasted in GenBank, and the genus is confirmed for each species, and the accession numbers are provided in Table 1.

**Table 1.** Voucher specimen information

Species	GenBank accession number	Sequence length	Collection localities
*Platocoelotes luoi* sp. n.	KR065578	638 bp	Ciping Village, Jinggangshan City, Jiangxi Province, China
*Platocoelotes qinglinensis* sp. n.	KR065579	557 bp	Qinglin Village, Daguang County, Zhaotong City, Yunnan Province, China
*Platocoelotes shuiensis* sp. n.	KR065580	638 bp	Yushexianggantang Village, Shuicheng County, Liupanshui City, Guizhou Province, China
*Platocoelotes tianyangensis* sp. n.	KR065581	629 bp	Pingzhai Village, Xingwen County, Yibin City, Sichuan Province, China
*Platocoelotes xianwuensis* sp. n.	KR065577	629 bp	Xiejiaba Village, Xuanen County, Hubei Province, China

All of the specimens (including molecular vouchers) are deposited in the Institute of Zoology, Chinese Academy of Sciences in Beijing (IZCAS).

## Taxonomy

### Family Agelenidae C.L. Koch, 1837 Subfamily Coelotinae F.O.P.-Cambridge, 1893

#### Genus Platocoelotes

Taxon classificationAnimaliaAraneaeAgelenidae

Wang, 2002

Platocoelotes : Wang 2002: 122. Type species *Coelotes impletus* Peng & Wang, 1997, from Hunan, China.

##### Diagnosis.

**Figure 1.**
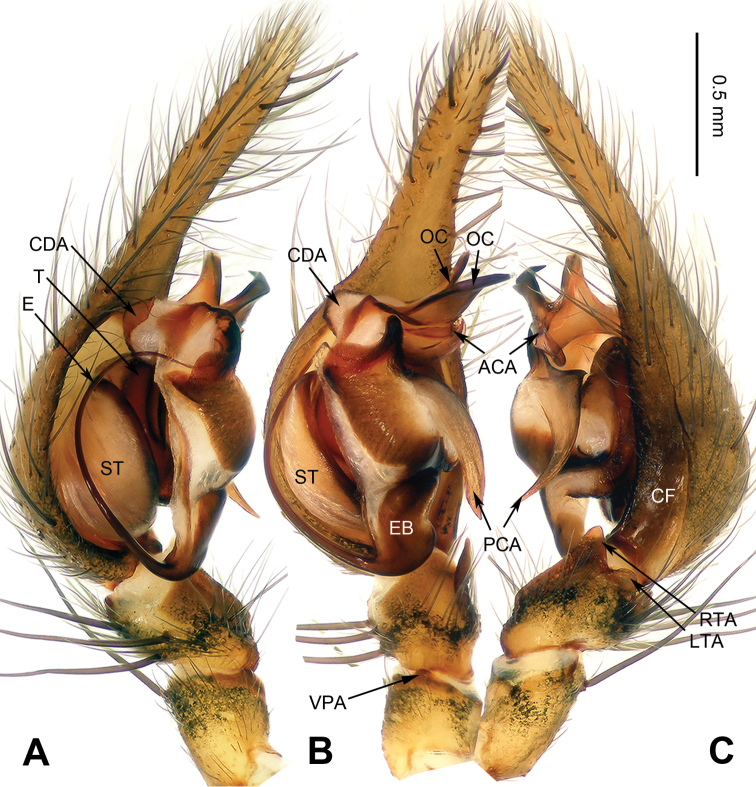
*Platocoelotes luoi* sp. n., holotype male. **A** Left palp, prolateral view **B** Left palp, ventral view **C** Left palp, retrolateral view. ACA = anterior conductor apophysis; CDA = dorsal conductor apophysis; CF = cymbial furrow; LTA = dorso-retrolateral tibial apophysis; OC = outgrowth in anterior conductor apophysis; PCA = posterior conductor apophysis; RTA = retrolateral tibial apophysis; VPA = ventral patellar apophysis. Scale bar: Equal for **A, B, C**.

**Figure 2.**
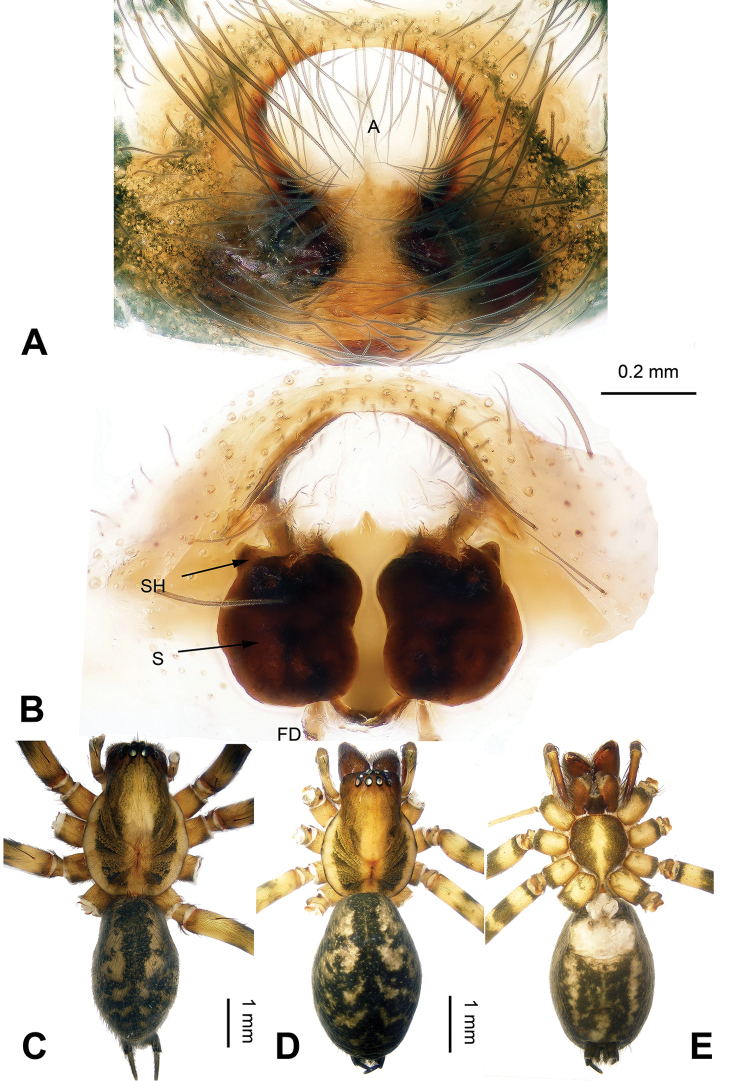
*Platocoelotes luoi* sp. n., one paratype female. **A** Epigyne, ventral view **B** Vulva, dorsal view **C** Male habitus, dorsal view. **D** Female habitus, dorsal view **E** Female habitus, ventral view. A = epigynal atrium; FD = fertilization duct; S = spermathecae; SH = spermathecal head. Scale bars: Equal for **A, B**, equal for **C, D, E**.

Male palp with two conductor apophyses (anterior conductor apophysis and posterior conductor apophysis) (Fig. 1B); only one conductor apophysis in other similar genera. There are two patellar apophyses (one or both of them are highly reduced in size in some species) in *Platocoelotes* species; other coelotines usually have only one. The female can be distinguished from other coelotines by the large epigynal atrium, the absence of epigynal teeth, simple spermathecae and indistinct copulatory ducts (Fig. 2A–B).

#### Platocoelotes luoi

Taxon classificationAnimaliaAraneaeAgelenidae

Chen & Li sp. n.

http://zoobank.org/689CE1F6-D99C-4794-A6E1-4AB8A0054FDA

Figs 1 , 2 , 11

##### Type material.

**Holotype** ♂: China: Jiangxi: Jinggangshan City: Ciping Village, Shiyan Cave, N26°36'11", E114°12'46", elevation: 977 m, 3.I.2013, Y.C. Li. **Paratypes**: 5♀, same data as holotype; 2♀, China: Jiangxi: Jinggangshan City: Ciping Village, Shiyan Cave, N26°36'11", E114°12'46", elevation: 977 m, 4.V.2013, Y.F. Luo and J.C. Liu.

##### Etymology.

The specific name is a patronym in honor of the collector Yufa Luo; noun (name) in genitive case.

##### Diagnosis.

The male can be distinguished from all other *Platocoelotes* by the distinct dorsal conductor apophysis and two sheet-shaped outgrowths of the anterior conductor apophysis (Fig. 1A–C). The female can be distinguished from all of the other *Platocoelotes*, except *Platocoelotes globosus* Xu & Li 2008, by having a rounded epigynal atrium and can be distinguished from *Platocoelotes globosus* by anteriorly located epigynal hoods and distinct spermathecal heads (Fig. 2A–B; Xu and Li 2008: figs 9–10).

##### Description.

**Male (holotype)**: Total length 7.92. Carapace 3.92 long, 2.80 wide. Abdomen 4.00 long, 2.36 wide. Eye sizes and interdistances: AME 0.16, ALE 0.22, PME 0.19, PLE 0.20; AME-AME 0.08, AME-ALE 0.03, PME-PME 0.13, PME-PLE 0.15. Leg measurements: I: 17.37 (4.49, 5.51, 4.61, 2.76); II: 15.45 (4.10, 4.81, 4.10, 2.44); III: 14.07 (3.72, 4.33, 3.97, 2.05); IV: 19.42 (4.87, 5.77, 5.96, 2.82). Chelicerae with three promarginal and two retromarginal teeth. Palp: patellar apophysis absent, ventral patellar apophysis short; RTA with pointed tip; LTA long, about 1/2 length of RTA; cymbial furrow long, about 1/3 length of cymbium; anterior conductor apophysis short; posterior conductor apophysis long, about 1/2 length of cymbium; dorsal conductor apophysis present, with two distinct apophyses; embolus filiform, with pointed tip (Fig. 1A–C).

**Female (one of paratypes)**: Total length 9.25. Carapace 4.80 long, 2.80 wide. Abdomen 4.45 long, 3.00 wide. Eye sizes and interdistances: AME 0.14, ALE 0.19, PME 0.16, PLE 0.20; AME-AME 0.06, AME-ALE0.03, PME-PME 0.07, PME-PLE 0.10. Leg measurements: I: 12.10 (3.20, 4.10, 2.85, 1.95); II: 10.80 (2.90, 3.55, 2.60, 1.75); III: 9.60 (3.10, 3.15, 2.10, 1.25); IV: 13.05 (3.65, 4.20, 3.45, 1.75). Chelicerae with three promarginal and two retromarginal teeth. Epigyne: atrium medium-sized, occupying 1/3 of epigyne; hoods absent; spermathecae simple, spermathecal heads small, located anteriorly; copulatory ducts indistinct; fertilization ducts widely separated by at least their width (Fig. 2A–B).

##### Distribution.

Known only from the type locality (Fig. 11).

#### Platocoelotes qinglinensis

Taxon classificationAnimaliaAraneaeAgelenidae

Chen & Li sp. n.

http://zoobank.org/ECCCEC68-EFB6-45D8-9434-483E4C518677

Figs 3 , 4 , 11

##### Type material.

**Holotype** ♂: China: Yunnan: Zhaotong City: Daguang County: Mohan Town, Qinglin Village, Qinglong Cave, N27°41'37", E103°44'52", elevation: 1289 m, 18.III.2014, Y.C. Li & J.C. Liu. **Paratypes**: 1♀1♂, same data as holotype.

##### Etymology.

The specific name refers to the type locality; adjective.

##### Diagnosis.

**Figure 3.**
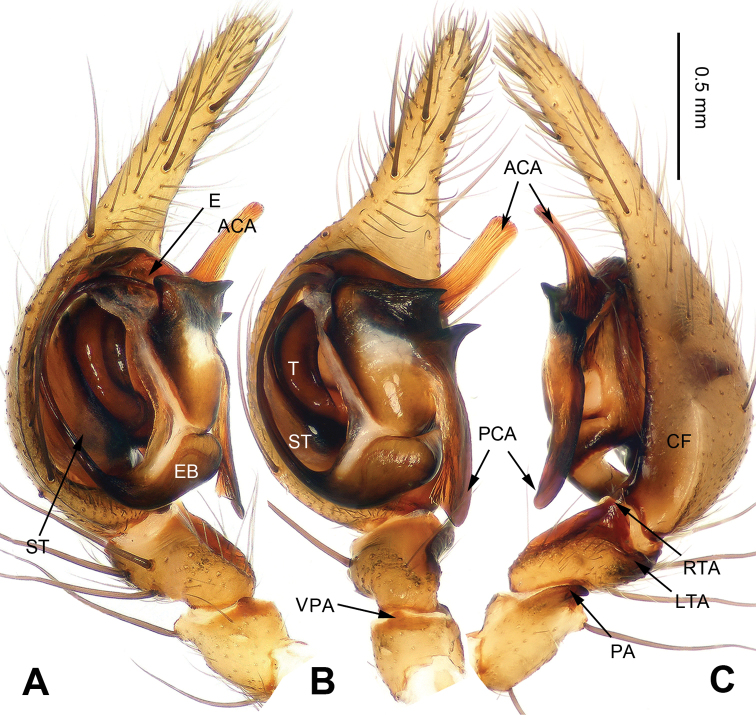
*Platocoelotes qinglinensis* sp. n., holotype male. **A** Right palp (inverted), prolateral view **B** Right palp (inverted), ventral view **C** Right palp (inverted), retrolateral view. ACA = anterior conductor apophysis; CF = cymbial furrow; LTA = dorso-retrolateral tibial apophysis; PA = patellar apophysis; PCA = posterior conductor apophysis; RTA = retrolateral tibial apophysis; VPA = ventral patellar apophysis. Scale bar: Equal for **A, B, C**.

**Figure 4.**
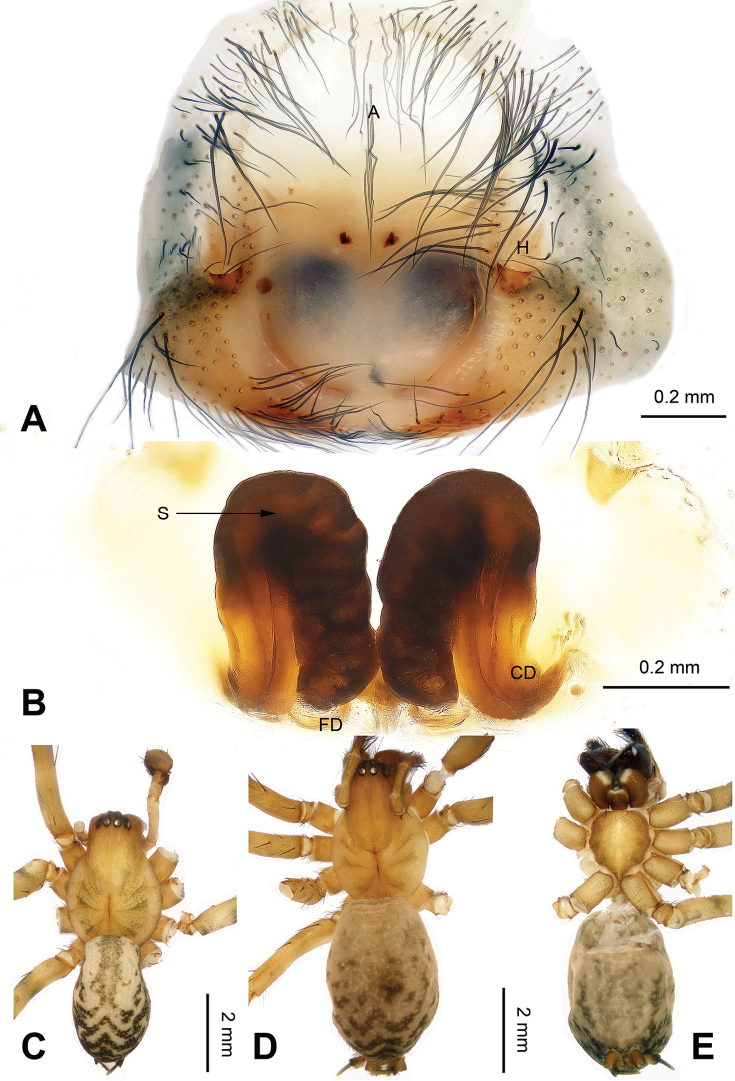
*Platocoelotes qinglinensis* sp. n., paratype female. **A** Epigyne, ventral view **B** Vulva, dorsal view. **C** Male habitus, dorsal view **D** Female habitus, dorsal view **E** Female habitus, ventral view. A = epigynal atrium; CD = copulatory duct; FD = fertilization duct; H = epigynal hood; S = spermatheca. Scale bars: Equal for **C, D, E**.

The male can be distinguished from all other *Platocoelotes* species, except *Platocoelotes ampulliformis* Liu & Li, 2008, *Platocoelotes brevis* Liu & Li, 2008, *Platocoelotes latus* Xu & Li, 2008, *Platocoelotes paralatus* Xu & Li, 2008 and *Platocoelotes strombuliformis* Liu & Li, 2008, by having a thinner anterior conductor apophysis and can be distinguished from these five species by the presence of a broader cavity on the anterior conductor apophysis (Fig. 3A–C). The female can be distinguished from other *Platocoelotes* species, except *Platocoelotes latus*, by the large epigynal atrium and the medially situated epigynal hoods and can be distinguished from *Platocoelotes latus* by the distinct copulatory ducts, the absence of spermathecal heads, and the spermathecae together with the copulatory ducts, looks like an M (Fig. 4B; Xu and Li 2008: fig. 16).

##### Description.

**Male (holotype)**: Total length 7.35. Carapace 3.45 long, 2.35 wide. Abdomen 3.90 long, 2.75 wide. Eye sizes and interdistances: AME 0.18, ALE 0.23, PME 0.19, PLE 0.21; AME-AME 0.06, AME-ALE 0.03, PME-PME 0.09, PME-PLE 0.10. Leg measurements: I: 12.85 (3.60, 4.30, 3.00, 1.95); II: 10.80 (3.10, 3.50, 2.50, 1.70); III: 9.80 (2.75, 3.05, 2.50, 1.50); IV: 16.45 (4.00, 3.90, 3.81, 1.80). Chelicerae with three promarginal and two retromarginal teeth. Palp: patellar apophysis long, its length almost equal to patellar width; ventral patellar apophysis short, with rounded tip; RTA with pointed tip, slightly extending beyond distal margin of tibia; LTA short, approximately less than 1/5 length of RTA; cymbial furrow about 1/3 length of cymbium; conductor with long, canoe-like, blunt tip; posterior conductor apophysis long, about 1/2 length of cymbium; dorsal conductor apophysis absent; embolus filiform, beginning at 6-o’clock position, forming a semicircular shape (Fig. 3A–C).

**Female (paratype)**: Total length 6.41. Carapace 3.33 long, 2.56 wide. Abdomen 3.08 long, 1.92 wide. Eye sizes and interdistances: AME 0.19, ALE 0.18, PME 0.16, PLE 0.18; AME-AME 0.05, AME-ALE 0.04, PME-PME 0.07, PME-PLE 0.10. Leg measurements: I: 15.86 (4.10, 5.26, 4.05, 2.45); II: 13.75 (3.50, 4.45, 3.60, 2.20); III: 12.25 (3.25, 3.60, 3.65, 1.75); IV: 16.45 (4.25, 4.90, 4.95, 2.35). Chelicerae with three promarginal and two retromarginal teeth. Epigyne: atrium large, occupying 3/4 of epigyne; hoods distinct, located mediolaterally on epigynal plate; copulatory ducts broad; spermathecae simple; spermathecae together with the copulatory ducts, M-shape (Fig. 4A–B).

##### Distribution.

Known only from the type locality (Fig. 11).

#### Platocoelotes shuiensis

Taxon classificationAnimaliaAraneaeAgelenidae

Chen & Li sp. n.

http://zoobank.org/7A08050F-C46B-4F5A-8220-9130DFE25F48

Figs 5 , 6 , 11

##### Type material.

**Holotype** ♂: China: Guizhou: Liupanshui City: Shuicheng County: Yushexianggantang Village, Wuming Cave, N26°25'35", E104°48'55", elevation: 1345 m. 28.III.2013, H.F. Zhao and J.C. Liu. **Paratypes**: 10♀2♂, same data as holotype.

##### Etymology.

The specific name refers to the type locality; adjective.

##### Diagnosis.

**Figure 5.**
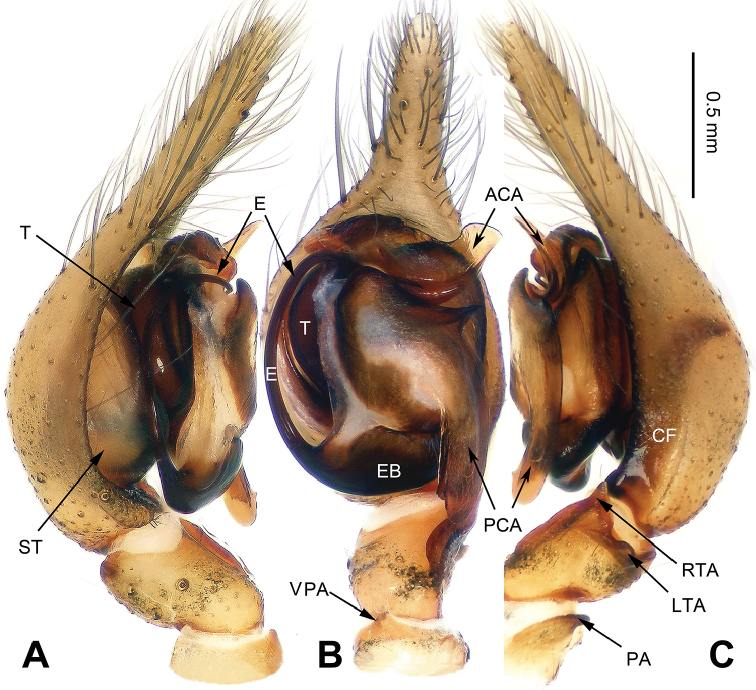
*Platocoelotes shuiensis* sp. n., holotype male. **A** Left palp, prolateral view **B** Left palp, ventral view **C** Left palp, retrolateral view. ACA = anterior conductor apophysis; CF = cymbial furrow; LTA = dorso-retrolateral tibial apophysis; PA = patellar apophysis; PCA = posterior conductor apophysis; RTA = retrolateral tibial apophysis; VPA = ventral patellar apophysis. Scale bar: Equal for **A, B, C**.

**Figure 6.**
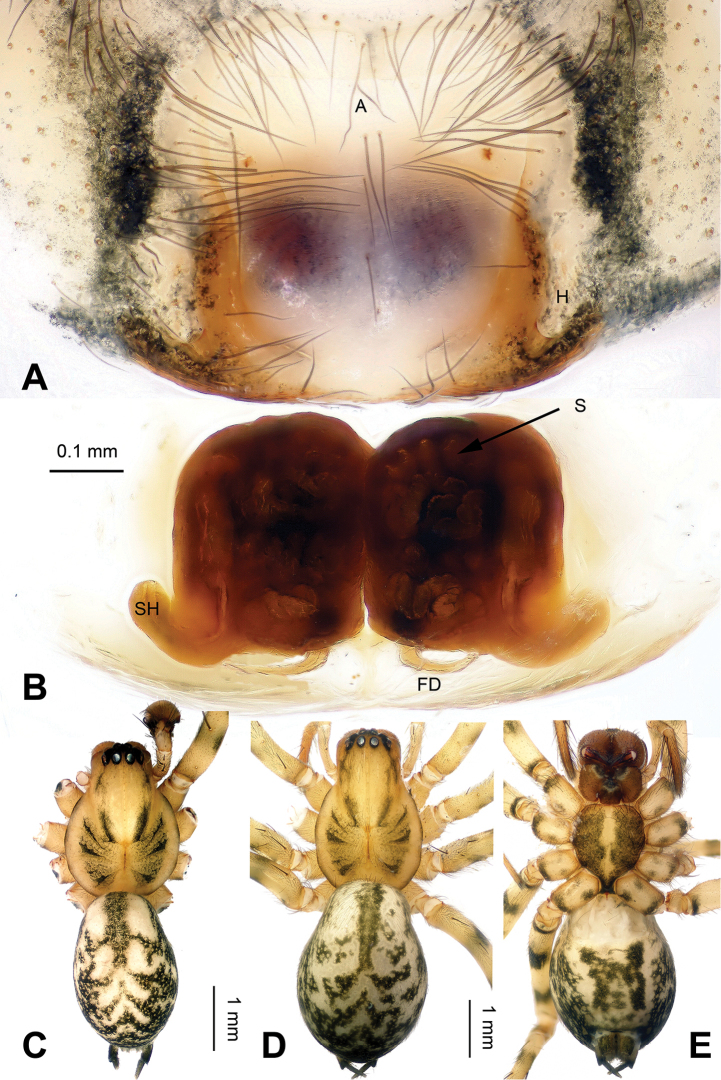
*Platocoelotes shuiensis* sp. n., one paratype female. **A** Epigyne, ventral view **B** Vulva, dorsal view **C** Male habitus, dorsal view **D** Female habitus, dorsal view **E** Female habitus, ventral view. A = epigynal atrium; FD = fertilization duct; H = epigynal hood; S = spermatheca; SH = spermathecal head. Scale bars: Equal for **A, B**; Equal for **C, D, E**.

The male can be distinguished from all other *Platocoelotes* species, except *Platocoelotes ampulliformis*, *Platocoelotes brevis*, *Platocoelotes latus*, *Platocoelotes paralatus*, *Platocoelotes qinglinensis* sp. n. and *Platocoelotes strombuliformis*, by having a slender anterior conductor apophysis and a long posterior conductor apophysis and can be distinguished from these six species by the anterior conductor apophysis being concave mesally (Fig. 5A–C). The female can be distinguished from all other *Platocoelotes* species, except *Platocoelotes latus*, by having a large epigynal atrium and can be distinguished from *Platocoelotes latus* by the posteriorly situated epigynal hoods and twined spermathecae, forming quadrate structure (Fig. 6B; Xu and Li 2008: figs 15–16).

##### Description.

**Male (holotype)**: Total length 4.75. Carapace 2.45 long, 2.15 wide. Abdomen 2.30 long, 1.50 wide. Eye sizes and interdistances: AME 0.17, ALE 0.15, PME 0.19, PLE 0.16; AME-AME 0.08, AME-ALE 0.03, PME-PME 0.06, PME-PLE 0.12. Leg measurements: I: 11.46 (2.81, 3.92, 2.81, 1.92); II: 9.44 (2.40, 3.20, 2.24, 1.60); III: 9.28 (2.56, 2.80, 2.40, 1.52); IV: 12.32 (3.28, 3.68, 3.52, 1.84). Chelicerae with three promarginal and two retromarginal teeth. Palp: patellar apophysis long; ventral patellar apophysis short, with blunt tip; RTA with pointed tip extending slightly beyond distal margin of tibia; dorso-retrolateral tibial apophysis short, about 1/3 length of RTA; cymbial furrow about 1/4 length of cymbium; anterior conductor apophysis long, approximately 1/3 length of cymbium, with blunt tip; posterior conductor apophysis long, about twice the length of cymbial furrow; dorsal conductor apophysis undeveloped; embolus filiform, arising at 6-o’clock position, forming a semicircle (Fig. 5A–C).

**Female (one of paratypes)**: Total length 5.96. Carapace 2.96 long, 2.80 wide. Abdomen 3.00 long, 2.04 wide. Eye sizes and interdistances: AME 0.13, ALE 0.14, PME 0.14, PLE 0.13; AME-AME 0.08, AME-ALE 0.04, PME-PME 0.07, PME-PLE 0.04. Leg measurements: I: 8.84 (2.38, 2.66, 2.20, 1.60); II: 7.57 (2.19, 2.50, 1.75, 1.24); III: 6.83 (1.80, 2.10, 1.73, 1.20); IV: 9.36 (2.50, 3.00, 2.50, 1.36). Epigyne: atrium large, occupying 4/5 of epigyne; hoods situated posteriorly, near the lateral atrial margins; spermathecae simple, convoluted, forming a square; spermathecal heads medium-sized, situated posteriorly and widely separated from each other; copulatory ducts absent (Fig. 6A–B).

##### Distribution.

Known only from the type locality (Fig. 11).

#### Platocoelotes tianyangensis

Taxon classificationAnimaliaAraneaeAgelenidae

Chen & Li sp. n.

http://zoobank.org/997FEC88-C19B-459D-904C-0A7258B26BF7

Figs 7 , 8 , 11

##### Type material.

**Holotype** ♂: China: Sichuan: Yibin City: Xingwen County: Shihaidong, Pingzhai Village, Tianyang Cave, N28°11'46", E105°8'24", elevation: 835 m. 16.XII.2014, Y.C. Li and Z. G. Chen. **Paratypes**: 16♀5♂, same data as holotype; 1♀: China: Sichuan: Yibin City: Xingwen County: Shihaidong, Pingzhai Village, Tianyang Cave, N28°11'46", E105°8'24", elevation: 835 m. 25.IV.2014, Y.C. Lin, H.F. Zhao, Y.C. Li, F.Y. Li and J.L. Wu.

##### Etymology.

The specific name refers to the type locality; adjective.

##### Diagnosis.

**Figure 7.**
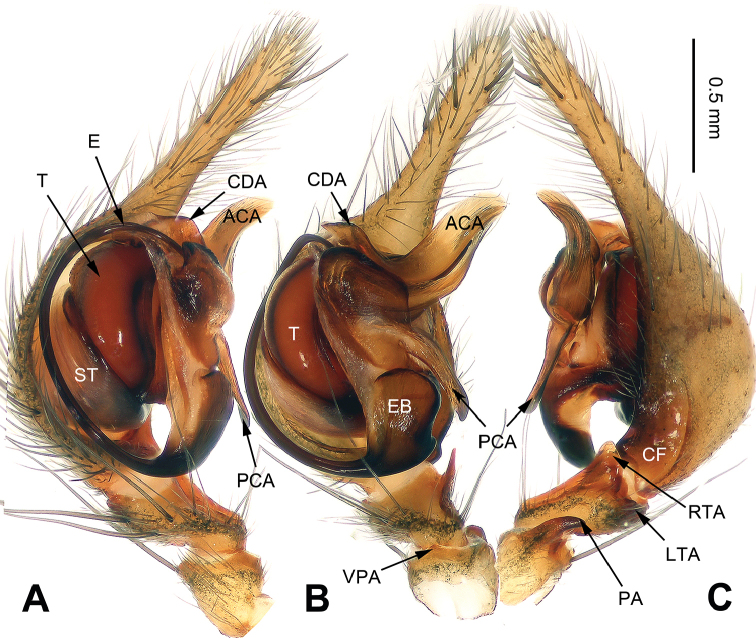
*Platocoelotes tianyangensis* sp. n., holotype male. **A** Left palp, prolateral view **B** Left palp, ventral view **C** Left palp, retrolateral view. ACA = anterior conductor apophysis; CDA = dorsal conductor apophysis; CF = cymbial furrow; LTA = dorso-retrolateral tibial apophysis; PA = patellar apophysis; PCA = posterior conductor apophysis; RTA = retrolateral tibial apophysis; VPA = ventral patellar apophysis. Scale bars: Equal for **A, B, C**.

**Figure 8.**
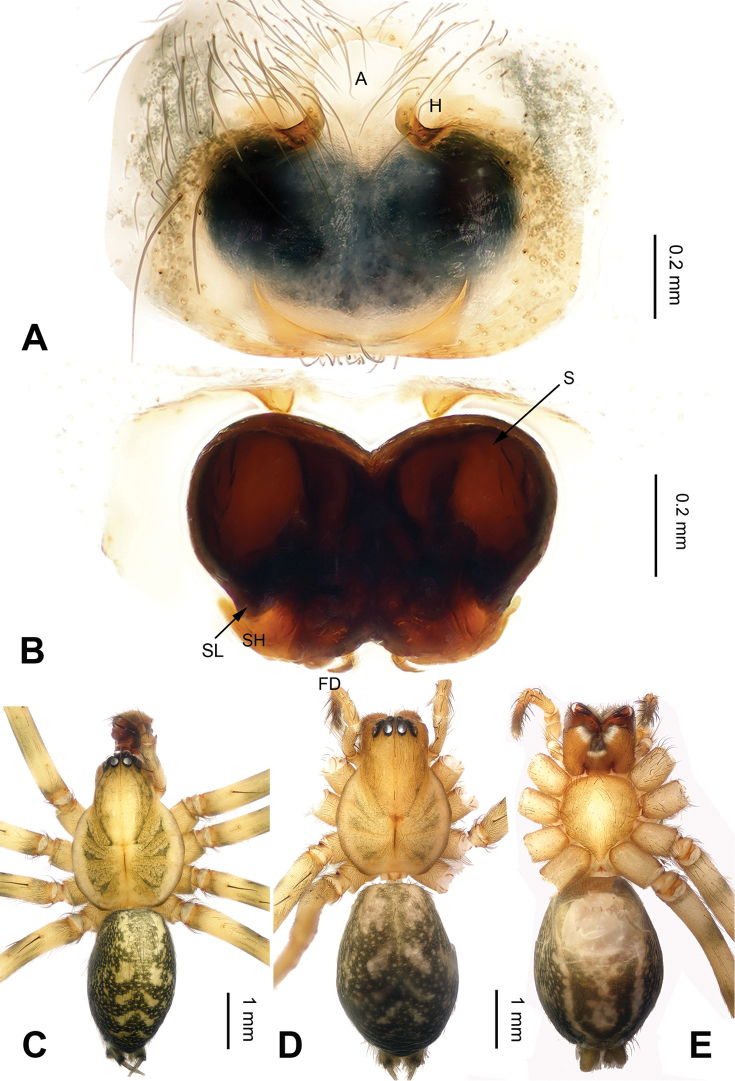
*Platocoelotes tianyangensis* sp. n., one paratype female. **A** Epigyne, ventral view **B** Vulva, dorsal view **C** Male habitus, dorsal view **D** Female habitus, dorsal view **E** Female habitus, ventral view. A = epigynal atrium; FD = fertilization duct; H = epigynal hood; S = spermatheca; SH = spermathecal head; SL = spermathecal lobe. Scale bars: Equal for **C, D, E**.

The male can be distinguished from all of the other *Platocoelotes* species, except *Platocoelotes ampulliformis*, *Platocoelotes brevis*, *Platocoelotes latus*, *Platocoelotes paralatus*, and *Platocoelotes strombuliformis*, by having a thinner anterior conductor apophysis and a longer posterior conductor apophysis and can be distinguished from these five species by the large tegulum and broader distal end of the anterior conductor apophysis (Fig. 7A–C). The female can be distinguished from all of the other *Platocoelotes* species, except *Platocoelotes ampulliformis*, by the presence of a small anterior epigynal atrium and a large posterior epigynal atrium (Fig. 8A; Liu and Li 2008: fig. 1E), and can be distinguished from *Platocoelotes ampulliformis* by fused spermathecae and the absence of copulatory ducts (Fig. 8B; Liu and Li 2008: fig. 1F).

##### Description.

**Male (holotype)**: Total length 5.44. Carapace 2.80 long, 2.16 wide. Abdomen 2.64 long, 1.66 wide. Eye sizes and interdistances: AME 0.15, ALE 0.17, PME 0.16, PLE 0.16; AME-AME 0.03, AME-ALE 0.02, PME-PME 0.08, PME-PLE 0.06. Leg measurements: I: 14.85 (3.80, 4.65, 3.90, 2.50); II: 12.20 (3.20, 3.75, 3.25, 2.00); III: 10.84 (2.92, 3.00, 3.16, 1.76); IV: 15.42 (3.92, 4.45, 4.65, 2.40). Chelicerae with three promarginal and two retromarginal teeth. Palp: patellar apophysis long; ventral patellar apophysis short, with blunt tip; RTA with pointed tip extending slightly beyond distal margin of tibia; LTA short, about 1/3 length of RTA; cymbial furrow short, about 1/5 length of cymbium; anterior conductor apophysis broad and long, with blunt tip; posterior conductor apophysis thin, shorter than anterior conductor apophysis, length subequal to cymbial furrow (Fig. 7A–C).

**Female (one of paratypes)**: Total length 5.77. Carapace 2.82 long, 1.92 wide. Abdomen 2.95 long, 2.10 wide. Eye sizes and interdistances: AME 0.09, ALE 0.16, PME 0.17, PLE 0.20; AME-AME 0.06, AME-ALE 0.05, PME-PME 0.07, PME-PLE 0.09. Leg measurements: I: 10.80 (2.88, 3.50, 2.56, 1.86); II: 9.03 (2.56, 2.88, 2.05, 1.54); III: 8.21 (2.44, 2.56, 1.99, 1.22); IV: 11.10 (2.89, 3.35, 3.21, 1.47). Epigyne: atrium large, occupying 1/2 of epigynal plate; hoods located in the anterior part of epigyne, near each other; spermathecae simple and medially fused to each other; spermathecal stalks broad; spermathecal heads small, located at posterior part of spermathecae; copulatory ducts absent. (Fig. 8A–B).

##### Distribution.

Known only from the type locality (Fig. 11).

#### Platocoelotes xianwuensis

Taxon classificationAnimaliaAraneaeAgelenidae

Chen & Li sp. n.

http://zoobank.org/BC80792A-D0A5-46A8-A86D-F58263B3315B

Figs 9 , 10 , 11

##### Type material.

**Holotype** ♂: China: Hubei: Enshi Prefecture: Xuanen County: Zhushan Town, Park, Xiejiaba Village, Xianwu Cave, N29°57'06", E109°29'49", elevation 853 m., 14.XII.2014, Y.C. Li and Z.G. Chen. **Paratypes**: 16♀5♂, same data as holotype; 1♀, China: Hubei: Enshi Prefecture: Xuanen County: Zhushan Town, Park, Xiejiaba Village, Xianwu Cave, N29°57'06", E109°29'49", elevation 853 m., 18.I.2014, Y.C. Li and J.C. Liu.

##### Etymology.

The specific name refers to the type locality; adjective.

##### Diagnosis.

**Figure 9.**
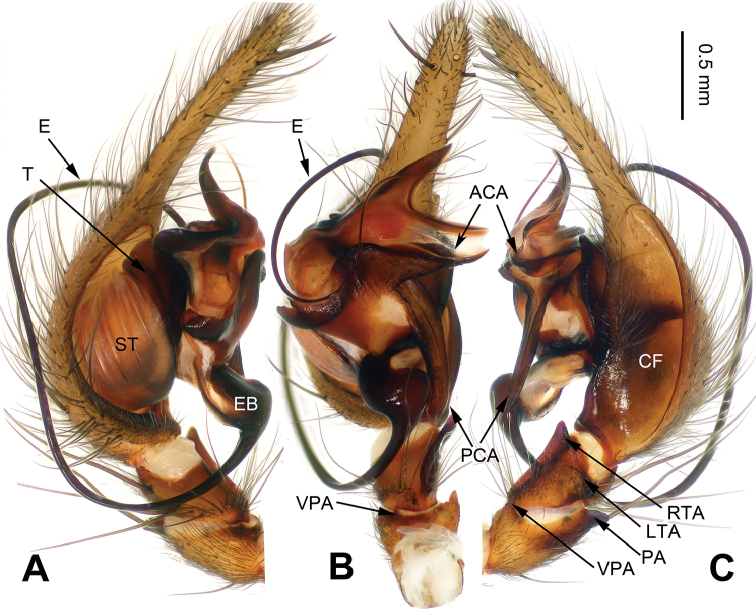
*Platocoelotes xianwuensis* sp. n., holotype male. **A** Left palp, prolateral view **B** Left palp, ventral view **C** Left palp, retrolateral view. ACA = anterior conductor apophysis; CDA = dorsal conductor apophysis; CF = cymbial furrow; LTA = dorso-retrolateral tibial apophysis; PA = patellar apophysis; PCA = posterior conductor apophysis; RTA = retrolateral tibial apophysis; VPA = ventral patellar apophysis. Scale bar: Equal for **A, B, C**.

**Figure 10.**
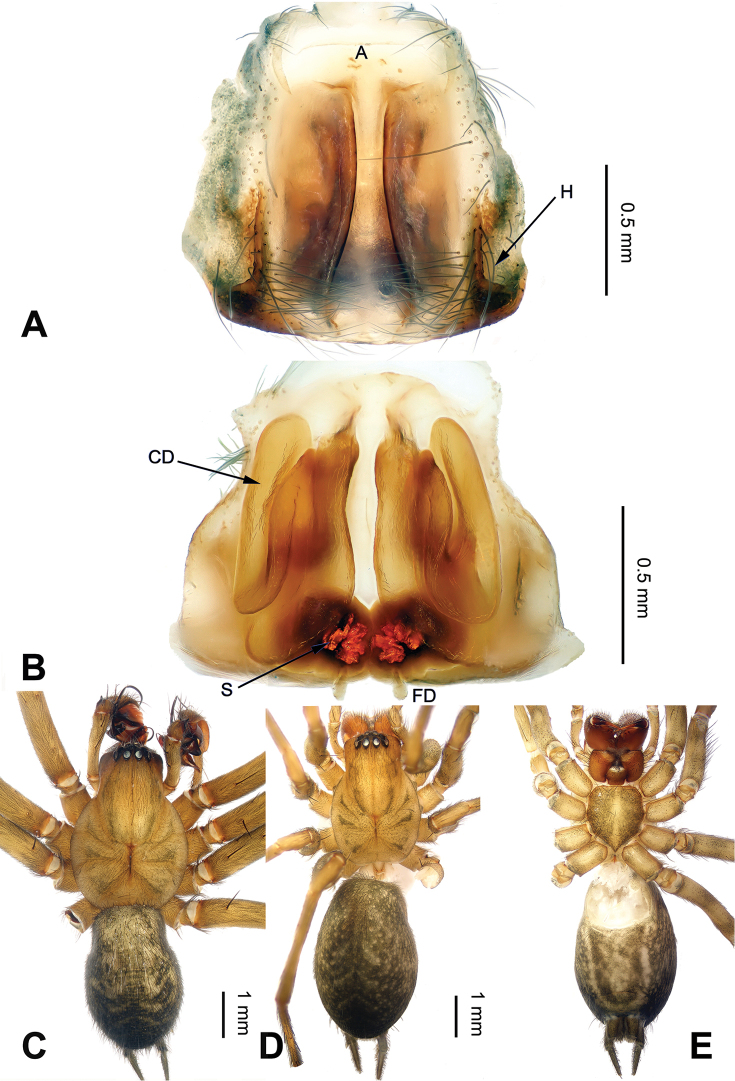
*Platocoelotes xianwuensis* sp. n., one paratype female. **A** Epigyne, ventral view **B** Vulva, dorsal view **C** Male habitus, dorsal view **D** Female habitus, dorsal view **E** Female habitus, ventral view. A = epigynal atrium; CD = copulatory duct; FD = fertilization duct; H = epigynal hood; S = spermatheca. Scale bars: Equal for **C, D, E**.

The male can be distinguished from all other *Platocoelotes* species, except *Platocoelotes icohamatoides* Peng & Wang, 1997, *Platocoelotes impletus* Peng & Wang, 1997, *Platocoelotes lichuanensis* Chen & Zhao, 1998 and *Platocoelotes kailiensis* Wang, 2003, by the presence of a branch in the anterior conductor apophysis and can be distinguished from these four species by the long anterior conductor branch, with a wide base that is spiky distally, and the hyaline part in the middle of the anterior conductor apophysis (Fig. 9A–C). The female can be distinguished from all other *Platocoelotes* species, except *Platocoelotes impletus* and *Platocoelotes icohamatoides*, by the presence of a long, narrow epigynal septum and can be distinguished from *Platocoelotes impletus* by the rectangular epigynal atrium, and the longer, thinner copulatory ducts (Fig. 10A–B; Wang 2003: fig. 75A–B). It can be distinguished from *Platocoelotes icohamatoides* by having fewer loops in the copulatory ducts (with 2 loops) (Fig. 10B; Wang 2003: fig. 76B).

##### Description.

**Male (holotype)**: Total length 7.50. Carapace 4.10 long, 3.05 wide. Abdomen 3.40 long, 2.45 wide. Eye sizes and interdistances: AME 0.19, ALE 0.25, PME 0.19, PLE 0.20; AME-AME 0.05, AME-ALE 0.03, PME-PME 0.11, PME-PLE 0.14. Leg measurements: I: 19.41 (5.19, 6.47, 4.93, 2.82); II: 16.85 (4.68, 5.38, 4.29, 2.50); III: 15.31 (4.29, 4.48, 4.36, 2.18); IV: 20.44 (5.45, 6.15, 6.28, 2.56). Chelicerae with three promarginal and two retromarginal teeth. Palp: patellar apophysis long; ventral patellar apophysis short, about 1/5 length of patellar apophysis, with pointed tip; RTA with pointed tip, extending beyond the tibia; LTA short, about 1/5 length of RTA; cymbial furrow long, about 1/2 length of cymbium; anterior conductor apophysis long, with middle part hyaline, with pointed tip; posterior conductor apophysis long, subequal to the length of cymbial furrow; dorsal conductor apophysis absent; embolus filiform, elongate (Fig. 9A–C).

**Female (one of paratypes)**: Total length 7.80. Carapace 3.70 long, 2.25 wide. Abdomen 4.10 long, 2.45 wide. Eye sizes and interdistances: AME 0.15, ALE 0.20, PME 0.16, PLE 0.14; AME-AME 0.08, AME-ALE 0.03, PME-PME 0.09, PME-PLE 0.10. Leg measurements: I: 10.89 (3.14, 3.50, 2.60, 1.65); II: 9.75 (2.95, 3.25, 2.00, 1.55); III: 8.65 (2.50, 2.80, 2.25, 1.10); IV: 11.90 (3.25, 3.75, 3.15, 1.75). Epigyne: atrium medium size, occupying 1/4 of epigynal plate, anterior part of atrium oblong, posterior part longitudinally elongate, about 4/5 length of epigyne; epigynal hoods situated posteriorly, near the lateral atrial margin; spermathecae simple, located in posterior of epigyne; spermathecal head absent; copulatory ducts long, broad and looped (Fig. 10A–B).

##### Distribution.

**Figure 11.**
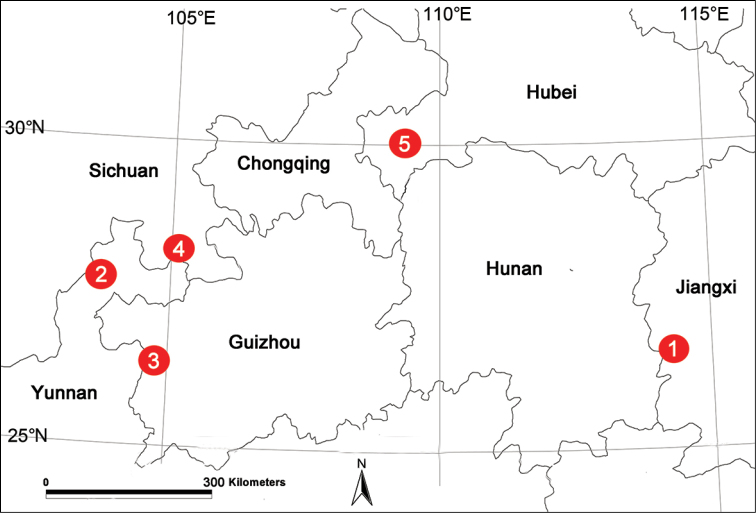
Localities of new *Platocoelotes* species from China. **1** *Platocoelotes luoi* sp. n. **2** *Platocoelotes qinglinensis* sp. n. **3** *Platocoelotes shuiensis* sp. n. **4** *Platocoelotes tianyangensis* sp. n. **5** *Platocoelotes xianwuensis* sp. n.

Known only from the type locality (Fig. 11).

## Supplementary Material

XML Treatment for Platocoelotes

XML Treatment for Platocoelotes luoi

XML Treatment for Platocoelotes qinglinensis

XML Treatment for Platocoelotes shuiensis

XML Treatment for Platocoelotes tianyangensis

XML Treatment for Platocoelotes xianwuensis
